# Increased HIV Incidence in *Wuchereria bancrofti* Microfilaria Positive Individuals in Tanzania

**DOI:** 10.3390/pathogens12030387

**Published:** 2023-02-28

**Authors:** Jonathan Mnkai, Manuel Ritter, Lucas Maganga, Leonard Maboko, Willyhelmina Olomi, Petra Clowes, Jessica Minich, Agola Eric Lelo, Daniel Kariuki, Alexander Yaw Debrah, Christof Geldmacher, Michael Hoelscher, Elmar Saathoff, Mkunde Chachage, Kenneth Pfarr, Achim Hoerauf, Inge Kroidl

**Affiliations:** 1 National Institute of Medical Research (NIMR), Mbeya Medical Research Center (MMRC), Mbeya P.O. Box 2410, Tanzania; 2 Institute for Medical Microbiology, Immunology and Parasitology (IMMIP), University Hospital Bonn (UKB), 53127 Bonn, Germany; 3 Tanzania Commission for AIDS, Dodoma P.O. Box 2904, Tanzania; 4 Kenya Medical Research Institute (KEMRI), KNH, Nairobi, Kenya; 5 College of Health Sciences, Jomo Kenyatta University of Agriculture and Technology (JKUAT), Juja, Kenya; 6 Kumasi Centre for Collaborative Research (KCCR), Kwame Nkrumah University of Science and Technology, UPO, PMB, Kumasi, Ghana; 7 Division of Infectious Diseases and Tropical Medicine, University Hospital of the University of Munich (LMU), 80802 Munich, Germany; 8 German Centre for Infection Research (DZIF), Partner Site Munich, 80802 Munich, Germany; 9 Mbeya College of Health and Allied Sciences (UDSM-MCHAS), University of Dar es Salaam, Mbeya 608, Tanzania; 10 German Centre for Infection Research (DZIF), Partner Site Bonn-Cologne, 53127 Bonn, Germany; 11 German-West African Centre for Global Health and Pandemic Prevention (G-WAC), Partner Site Bonn, 53127 Bonn, Germany

**Keywords:** HIV, incidence, lymphatic filariasis, microfilariae, immunomodulation

## Abstract

Background: Infections with *Wuchereria bancrofti* are associated with reduced immunity against concomitant infections. Indeed, our previous study described a 2.3-fold increased HIV incidence among individuals with *W. bancrofti* infection, as measured by the circulating filarial antigen of the adult worm. This new study aimed to retrospectively determine microfilariae status of the participants to assess if the previously described increased HIV susceptibility was associated with the presence of MF in the same cohort. Methods: CFA positive but HIV negative biobanked human blood samples (*n* = 350) were analyzed for *W. bancrofti MF* chitinase using real time PCR. Results: The PCR provided a positive signal in 12/350 (3.4%) samples. During four years of follow-up (1109 person years (PY)), 22 study participants acquired an HIV infection. In 39 PY of *W. bancrofti* MF chitinase positive individuals, three new HIV infections occurred (7.8 cases per 100 PY), in contrast to 19 seroconversions in 1070 PY of *W. bancrofti* MF chitinase negative individuals (1.8 cases per 100 PY, *p* = 0.014). Conclusions: In the subgroup of MF-producing Wb-infected individuals, the HIV incidence exceeded the previously described moderate increased risk for HIV seen in all Wb-infected individuals (regardless of MF status) compared with uninfected persons from the same area.

## 1. Introduction

Lymphatic filariasis (LF) is a neglected parasitic tropical disease that affects the lymphatic system and can result in lymphedema (elephantiasis) and edema of the scrotum (hydrocele). LF is a leading cause of long-term and permanent disability in the world [1]. The numbers of infected individuals have dropped during the last two decades due to large mass treatment campaigns coordinated by the Global Alliance for Eliminating Lymphatic Filariasis (GAELF). There was a reported reduction from 199 million cases globally in 2000 to 51 million infected individuals in [1,2]. *Wuchereria bancrofti* (*W. bancrofti*) accounts for 90% of all global LF cases and is reported to be responsible for all LF cases in Africa [1]. Adult filarial worms dwell in the lymphatic vessels and destroy the normal functioning of the lymphatic system. These worms may live for 10 to 12 years and produce millions of microfilariae (MF) circulating in the blood during their lifetime. Microfilaremia is used to determine levels of endemicity and can help assess pre- and post-mass drug administration (MDA) transmission [3,4]. The Tanzanian National Lymphatic Filariasis Elimination Programme (NLFEP) started MDA in 2000 in the coastal areas and in 2009 in the Mbeya region of Tanzania. Earlier publications had indicated a prevalence of 42.3% of LF in adult individuals in the Kyela district in southwestern Tanzania, using the measurement of circulating filarial antigen with the TropBio Og4C3 ELISA for prevalence estimates [5]. However, our recent publication, focusing on participants aged above 14 in the same region, reported an impressive reduction from 35.1% to 1.7% [6].

The majority of infected individuals remain asymptomatic, but appear to have a reduced immunity against viral, bacterial or other parasitic infections, with many of them having MF (immature larvae) in their peripheral blood [7,8]. Other individuals in the same endemic community have undetectable circulating filarial antigen or MF, but suffer from disabling clinical symptoms [4]. It has been reported that asymptomatic infections with *W. bancrofti* are associated with systematic activation of CD4+ T cells decreased Th2 response, and pronounced regulatory T and B cells and dominant IgG4 and IL-10 responses [8,9,10].

The detection of MF on thick blood smears on slides is used alongside other techniques for diagnosing LF. It necessitates expert and labor-intensive work, e.g., morphological identification of MF, and also requires blood to be collected at night when MF transmission occurs [11]. Since the 1980s, immunoassays have been developed that use monoclonal antibodies AD12 and Og4C3 directed against antigen circulating in the blood of Bancroftian filariasis patients, which are not restricted to the use of night blood [12,13]. Various authors have reported the use of molecular techniques, particularly polymerase chain reaction (PCR), in the diagnosis of filariasis in human samples [14,15,16]. To identify samples with MF, we established a real-time PCR for the detection of the *W. bancrofti* microfilariae chitinase gene. The specificity and sensitivity of the *W. bancrofti* microfilariae-specific chitinase primers were assessed using genomic DNA (gDNA) extracted from known MF positive samples. Gel electrophoresis yielded characteristic bands of 150 base pairs which coincided with the *W. bancrofti* chitinase plasmid control gene.

The prevalence of HIV in Tanzania at that time was described as 7% (THMIS 2007/8), while the Mbeya region had a prevalence of 9.2% [17]. Our study region bordered Malawi, a country, which had reported an HIV prevalence of 15% to 17% in 2005 [18]. Among our study participants, the HIV prevalence was 13.1%, with the usual age distribution.

The association of endemic infections, such as malaria and helminthiasis (including LF), with human immunodeficiency virus (HIV) infection has been previously reported [5,19,20,21]. Using circulating filarial antigen (CFA) as a diagnostic marker of filariasis, we previously reported that individuals infected with LF have a 2.3-fold increased risk of acquiring HIV [22]. However, in the initial large general population study, MF were not measured.

MF positive individuals experience suppressed immune responses, revealed by reduced levels of Interleukin (IL) 5, 6, 10 and 17, as well as Interferon (IFN)-γ and TNF-α, compared to MF negative Wb-infected individuals [7]. Therefore, we hypothesized that the reduced antiviral capacity of the immune system of microfilaremic individuals might lead to an augmented susceptibility for HIV infections, as compared to amicrofilaremic Wb-infected persons. The present study was carried out to retrospectively assess the prevalence of *W. bancrofti* MF using biobanked blood samples that were collected during the initial study, to complement previous evaluations. Throughout this paper, we will use the following case definitions: (1) uninfected individuals from the study area (defined by a negative TropBio Og4C3 ELISA), (2) *W. bancrofti*-infected (defined by a positive TropBio Og4C3 ELISA)—MF negative (defined by a negative MF chitinase PCR), and (3) *W. bancrofti*-infected (defined by a positive TropBio Og4C3 ELISA)—MF positive (defined by a positive MF chitinase PCR).

## 2. Materials and Methods

### 2.1. Study Design

A retrospective study was conducted to analyze previously collected and biobanked human blood samples from individuals living in Kyela district, close to Lake Nyasa, in the Mbeya region, Tanzania. The specimens had been collected for the EMINI (Evaluation and Monitoring of the Impact of New Interventions; http://www.mmrp.org/projects/cohort-studies/emini accessed on 23 February 2023) cohort study, which was conducted at the National Institute for Medical Research (NIMR)—Mbeya Medical Research Centre (MMRC) between 2007 and 2011, as previously described [5]. In this new evaluation, 2 mL of biobanked blood samples was used from 350 participants who were recruited during the initial EMINI visit in 2007. The individuals selected were HIV negative but CFA positive in 2007, demonstrating an ongoing filarial infection. Clinical symptoms had no impact on the selection process. Samples were transported to the Institute for Medical Microbiology Immunology and Parasitology (IMMIP) in Bonn, Germany, for molecular detection of the *W. bancrofti* MF chitinase gene.

### 2.2. HIV and Filarial Antigen Testing

Blood collected during the EMINI cohort study had been previously tested for HIV using HIV1/2 STAT-PAK (Chem-Bio Diagnostics Systems, Medford, NY, USA) at baseline and follow-up visits, whereby positive results were confirmed by ELISA (Greenscreen ULTRA HIV Ag-Ab, Bio Rad, Hercules, CA, USA) and discrepancies resolved by Western blot (MPD HIV Blot2.2, MP Biomedicals, Geneva, Switzerland) [23]. A semi-quantitative ELISA specific for circulating filarial antigen (TropBio Og4C3, Townsville, Australia) was used for the diagnosis of *W. bancrofti* infection. According to the manufacturer’s recommendation (August, 2021), Standard 2 should be used to define the cut-off for positivity. If Standard 2 OD is within +/− 10% of 0.35, this value should be used as the cut-off. However, for ELISA plates with Standard 2 OD not within range (± 10% of 0.35), a default cut-off OD = 0.30 for positivity should be used. Optical density (OD) was measured and cut-off values of <0.2, ≥0.2 to ≤0.3, and >0.3 were considered as negative, intermediate and positive, respectively [22].

### 2.3. DNA Isolation and Purification

DNA from biobanked human blood pellets was extracted using TRIZOL and 1-bromo-3-chloropropaine reagents by the Invitrogen protocol (ThermoFisher Scientific, Waltham, MA, USA). The organic phase, including the interphase layer, which remained after RNA isolation was used for the DNA isolation, to which 700 µL of 100% ethanol was added and the tubes were inverted and incubated at room temperature (RT) for 5 min to precipitate the DNA and then centrifuged at 2000× *g* at 4 °C for 5 min. The pellet was washed twice by re-suspending in 1 mL of 0.1 M sodium citrate, 10% ethanol (pH 8.5), incubated at RT for 30 min and then centrifuged for 5 min at 2000× *g* at 4 °C. The pellet was then re-suspended with 1.5 mL of 75% ethanol and incubated at RT for 10 min and then centrifuged for 5 min at 2000× *g* and 4 °C. The DNA pellet was air dried for 10 min, followed by resuspension in 600 µL of 8 mM NaOH (pH 9.0) to solubilize the DNA, and centrifugation at 12,000× *g* at 4 °C for 10 min. Finally, the supernatant containing DNA was transferred into a new tube and DNA concentration was measured by a NanoVue machine (ThermoFisher Scientific) and then stored at −20 °C.

### 2.4. W. bancrofti Microfilaria Chitinase qPCR

The one hundred fifty-one (151) bp fragment of the *W. bancrofti* chitinase (GenBank: AF250997.1) was amplified in QuantiNova Probe PCR master mix (QIAGEN, Hilden, Germany) using 10 nM 5′-AAACAGCGATTGGAGCAGCG-3′ and 5′-ACCATGTACACCCCGACACC-3′ forward and reverse primers (Microsynth, Balgach, Switzerland) and 75 µM chitinase TaqMan 6-Fam labelled probe (Biomers.net GmbH, Ulm, Germany) in a total reaction volume of 20 µL. Plasmid DNA containing the *W. bancrofti* endochitinase sequence was used as the positive control and nuclease-free water was used as the no template control. The amplification and detection processes were carried out in triplicate under the following conditions: 95 °C for 2 min as the initial denaturation step, followed by 45 cycles of 95 °C for 10 s, 62 °C for 30 s and 72 °C for 20 s using a RotorGene 6000 thermocycler (Corbett Research, Sydney, Australia). For analysis with Rotor-Gene Q Software (version 2.3.1.49, Qiagen), the threshold was set at 0.02 and outlier removal at 15%. Human beta actin (GenBank: NG_007992.1) was amplified to verify DNA extraction in a reaction volume of 20 µL of 1× HotStar Taq master mix (Qiagen, Hilden, Germany), final concentration of 10 µM forward (5′-GAT GAG ATT GGC ATG GCT TTA-3′) and 10 µM reverse (5′-AAC CGA CTG CTG GTG TCA CCT TC-3′) primers (Microsynth, Göttingen, Germany), 200 µM dNTPs, 1× SYBR Green (1:1000 diluted in DMSO, Roche, Mannheim, Germany), and 0.5 units of HotStar Taq polymerase in a RotorGene 6000 (Corbett Research, Cambridge, UK) thermocycler under the following conditions: 95 °C for 15 min as the initial denaturation step, followed by 45 cycles of 94 °C for 10 s, 58 °C for 20 s and 72 °C for 20 s with acquisition on the green (FAM) channel [24]. Specific amplification was checked using a melting curve from 72 °C to 95 °C, rising by 1 °C each step with 4 s between steps and acquired on the green (FAM) channel, and analyzed with Rotor-Gene Q Software (ver. 2.3.1.49, Qiagen) at a threshold of 0.3. All DNA samples were positive for human beta-actin PCR. A mouse interferon gamma (mIFN-y) PCR was performed in chitinase negative samples to rule out possible presence of PCR inhibitors as described [25]. All mIFN-y PCR runs were negative for PCR inhibitors in the isolated DNA.

### 2.5. Validation and Optimization of the Established Chitinase PCR Protocol

**Figure 1:**
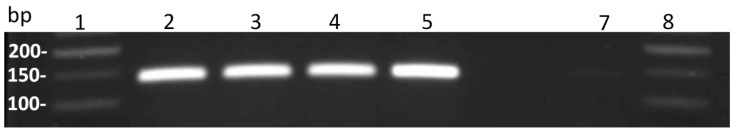
Optimized *W. bancrofti* MF chitinase PCR amplification. Base pair (bp); columns 1 and 8: 50 bp DNA ladder; columns 2–4: W. bancrofti (Wb) gDNA samples displaying single amplicons of 150 bp; column 5: Wb microfilariae (MF) chitinase plasmid positive control; column 7: no template control.

The performance of the *W. bancrofti* microfilariae-specific chitinase primers was assessed using gDNA extracted from known MF positive samples. CFA positive samples with known MF counts (CFA+/MF+) served as positive controls while samples from healthy individuals (CFA-/MF-) were selected as negative controls (Table 1). Gel electrophoresis yielded characteristic bands of 150 base pairs that coincided with the *W. bancrofti* chitinase plasmid (control gene) (Figure 1). In addition, DNA samples from individuals infected with other filariae (*Mansonella perstans*, *Loa loa* and *Onchocerca volvulus*) were used to evaluate specificity.

For sensitivity and specificity testing of the Wb chitinase PCR, three different sets of samples were used, one from West and one from Central Africa (Ghana and Cameroon, respectively), and another from the study area in Tanzania. Out of seven Ghanaian samples selected as positive controls (CFA and MF positive), six (85.7%) with >1500 MF/mL were positive for *W. bancrofti* MF chitinase, while one sample with only 200 MF/mL (14.3%) was negative (Table 2). Thirty CFA and MF negative blood samples from Ghana were used as negative controls. Twenty-nine of them (96.7%) were *W. bancrofti* microfilaria chitinase negative. In addition, 50 whole-blood samples from CFA negative study participants from the same village of the EMINI study in Tanzania were tested with the Wb MF chitinase PCR. All 50 samples tested negative. Furthermore, specificity of the *W. bancrofti* microfilaria chitinase PCR was confirmed by testing blood samples of individuals infected with other filarial nematodes (from Cameroon): *Mansonella perstans, Loa loa* and *Onchocerca volvulus*, which all tested negative. A summary of the results is shown in Table 1.

### 2.6. Statistical Analysis

Data were analyzed using Stata/SE software, version 17 (StataCorp, College Station, TX, USA) and GraphPad Prism software, version 8 (GraphPad Software Inc., La Jolla, CA, USA). Pearson’s Chi-square test was used to compare the binary outcomes between groups, while uni- and multivariable log link binomial regression was used to determine the association between potential risk factors and HIV incidence. Incidence rate ratios (IRR) with a 95% confidence interval (95% CI) were calculated and a *p*-value of <0.05 was considered statistically significant.

## 3. Results

### 3.1. Description of Study Participants

Biobanked blood samples of individuals from the Kyela village who had participated previously in the EMINI cohort study were used for these additional measurements. In Kyela, the EMINI study had enrolled individuals above the age of 14 in 2007 and annual follow-up visits were performed for five years. During each visit, which took place between 8 am and 2 pm, a blood sample was collected from each participant. Other data collected included socio-economic characteristics of the study participants, previous and current medical history, as well as individual knowledge regarding different diseases. Three hundred and fifty of the biobanked CFA positive samples of HIV-uninfected individuals were available and were used for Wb chitinase MF PCR assessment. Study participants’ ages ranged from 14 to 90 years with a median age of 32.7 years (IQR: 21.8–57 years), and 170 (48.6%) were female. All individuals were HIV negative at the time of enrollment and sample collection. The demographic data of participants for whom a sample was available were not significantly different from those whose samples were not available for testing (Table 1).

### 3.2. Quality Control, Optimization and Validation of W. bancrofti Microfilariae Chitinase PCR

The original aim had been to detect mRNA from microfilariae in blood. The chitinase is a gene expressed primarily in microfilariae [26]. Discrete transcripts encode multiple chitinase isoforms in Brugian microfilariae and would have been a good way to differentiate between microfilariae and mRNA from other stages [26]. As the PCR had been established, and it was very sensitive using blood samples spiked with plasmid containing the sequence and samples from Ghana used to work out the RNA extraction protocols, we continued using the real-time PCR for chitinase after we had determined that the Tanzanian samples would not provide RNA suitable for analysis.

Among the 350 analyzed blood samples, 12 (3.4%) tested positive for Wb microfilaria chitinase, with 9/170 (5.3%) positive results for samples from female participants and 3/180 (1.7%) from male participants (*p* = 0.062). After stratifying the cohort into three age groups, we did not find any differences in the proportion of Wb microfilaria chitinase between the groups. In adolescents aged 14 to <25 years, we found five of 117 (4.3%), in young adults of 25 to <45 years of age, three of 110 (2.7%), and in the group of individuals above 45 years of age, four of 123 (3.3%) were positive for *W. bancrofti* microfilaria chitinase (*p* = 0.803).

### 3.3. HIV Incidence in Wuchereria bancrofti Microfilariae Positive Individuals

During the four years of follow-up with the 350 Wb-infected persons, in 1109 person years (PY), 22 of the 350 individuals became infected with HIV (1.98 per 100 PY). Using a multivariable analysis to adjust for the influence of age, sex and socio-economic status, we observed 4.58 times higher incidence of HIV among the Wb MF chitinase positive individuals compared to the Wb-infected but MF negative individuals (*p* = 0.014, Table 3).

In a second step, we included individuals without Wb infection (CFA negative) from the previous EMINI study in the multivariable analysis. As MF can only be present in individuals harboring adult filarial worms, it seemed prudent to assume that all CFA negative individuals would be MF negative. To ensure this premise, we performed Wb chitinase PCR on 50 CFA negative samples from the Tanzanian study village and, indeed, all of them were PCR negative.

Known risk or preventive factors for HIV transmission, like “number of sex partners during the last year”, “condom use”, or “male circumcision”, were also incorporated [26,27]. The multivariable analysis showed that the incidence of HIV infection in Wb positive–MF negative individuals was twice as high as in CFA negative individuals. Moreover, Wb positive–MF positive individuals revealed an HIV incidence ~12 times higher than CFA negative individuals (Table 4).

The multivariable analysis also demonstrated a lower HIV incidence in men compared to women, in circumcised men compared to uncircumcised men, and in individuals with one sex partner during the last year compared to persons with two or more partners. All of this is known and proven in many studies.

## 4. Discussion

During an earlier study, we had observed a 2.3-times higher HIV incidence among those *W. bancrofti*-infected compared to filarial-uninfected individuals [22]. As we had focused on new infections for HIV, each HIV seroconversion was meticulously confirmed and the HIV positive status was verified with ELISA, Western blot and PCR. In addition, the HIV negative status of the previous year was corroborated [22,23]. In the previous study, an HIV incidence of 1.91 per 100 PY was found in filarial-infected versus 0.8 per 100 PY in filarial-uninfected participants (*p* = 0.03) [22]. Regrettably, we could not attribute the increased risk to microfilaremic or amicrofilaremic Wb-infected persons, as the microscopy of night blood was not performed during the previous study. As we know that microfilaremia is associated with dampened immune responses against filarial and other antigens, we strived to fill this gap [7]. Arndts et al. demonstrated an increase in IgE, along with a decrease in IL-6, IL-10, IFN-gamma and TNF-alpha in the blood of microfilaremic participants as compared to filarial-infected but amicrofilaremic individuals [7]. We hypothesized that these different immune responses would lead to a distinct impact on the susceptibility towards HIV of microfilaremic and amicrofilaremic Wb-infected individuals.

A PCR was developed that amplified a 151 bp fragment of the *W. bancrofti* chitinase gene. Evaluation of the Wb chitinase PCR on samples with a known number of Wb MF/mL, and on samples from individuals with other filarial infections, revealed a sensitivity and specificity of 85.7% and 99.2%, respectively. Testing of blood samples from participants with other filarial infections, namely, *O. volvulus, L. loa* and *M. perstans,* demonstrated high specificity of the Wb chitinase, as the results of those tests were all negative. In addition, samples of participants from the Tanzanian study area who were not infected with the adult worm of *W. bancrofti* (i.e., CFA negative)*,* displayed a negative Wb chitinase PCR test result. In contrast, six of the seven samples with known number of Wb microfilariae were positive. One sample with the lowest MF load (200 Mf/mL) was PCR negative.

Employing the Wb microfilaria chitinase PCR on the biobanked CFA positive blood samples identified twelve of the 350 (3.4%) samples as PCR positive. This percentage is much lower than we had expected, keeping in mind that the study was carried out in an area that was LF endemic at the time of sample collection and LF treatment programs had not started. However, as the larger initial study had been planned for other purposes, the samples were collected during the day, which might have reduced the numbers of MF in the peripheral blood substantially due to the nocturnal periodicity of the MF. In addition, treatment programs against river blindness, a disease caused by another filarial species, *O. volvulus*, had distributed ivermectin to the same population in the years before sample collection [27,28,29,30,31,32]. Similar to the situation in Tanzania, Endeshaw et al. published that the effect of ivermectin treatment for onchocerciasis in Ethiopia had lowered the MF prevalence, but not the overall *W. bancrofti* prevalence, as measured by adult worm antigen [33,34]. To correct for potential confounding factors, we performed a multivariable analysis. After adjusting for age, sex and socio-economic status, a 4.58-fold increased risk for HIV seroconversion was seen for microfilaremic participants, compared to filarial-infected amicrofilaremic individuals.

It is quite clear that only persons who harbor adult worms can have the offspring, the microfilariae, in their blood. With that knowledge, we included the data of known CFA negative individuals from the same study village into the analysis, who should be amicrofilaremic if the initial CFA test gave a true result. To ensure this, we randomly chose 50 CFA negative blood samples from participants within the study area and performed the Wb chitinase PCR and received only negative results. In addition, known risk factors for HIV transmission were included into the analysis to account for potential confounding factors. The multivariable analysis demonstrated an increased HIV susceptibility in people with more than two sex partners and a protective effect of male circumcision, which validated the quality of the analyzed data.

Regarding the filarial infection in the group of Wb-infected individuals, the amicrofilaremic individuals demonstrated a 2.1-times increased risk to acquire an HIV infection as compared to the Wb-uninfected individuals of the study village, whereas the microfilaremic individuals showed a 12.7-times increased risk (*p* < 0.001). We assume that the immune-suppressed state of microfilaremic Wb, as demonstrated by Arndts et al., lowers the antiviral capacity and increases the risk for transmission of viral infections, in this case HIV [7]. Support for this hypothesis is found in a recent study from Togo that showed that individuals infected with hookworms, another nematode species, have a higher viral load of HPV [35].

Our study had some limitations. We had to use a different definition of “helminth-positive” than in the previous publication [22]. Previously, we had only used the time period of stable infection with *W. bancrofti* to estimate the risk of new HIV infections. This is possible for the adult worm of *W. bancrofti,* which is not killed by either ivermectin or albendazole and therefore is detectable over several years. As we measured the CFA levels annually for five consecutive years, four “timespans” were created. The governmental treatment of LF started during the EMINI study, so “time spans” for MF were not useful, as MF are sensitive to ivermectin. In the analysis described now, we measured the MF in the blood only at one time point (the first survey of the previous study in 2007). We used the classifications of “ever infected with *W. bancrofti* with MF”, “ever infected with *W. bancrofti* without MF” and “never infected with *W. bancrofti*”.

We had tested this type of “ever infected” classification in the previous publication for *W. bancrofti* and obtained similar results as compared to the “conservative” description using stable time periods, which validated our modified classification [22]. Another limitation was the low number of Wb MF chitinase positive results. Initially, we had assumed that approximately half of the samples of *W. bancrofti*-infected individuals would be positive for MF. The low number of only 12 of 350 Wb MF chitinase positive cases was surprising. Due to logistical challenges in our previous study, the blood samples were collected during the day when MF numbers are absent or low due to MF nocturnal periodicity in peripheral blood, which could account for the low number of MF positive PCR test results [36]. It has been reported that Wb microfilaremia levels peak between 23:00 h and 01:00 h and decline to the lowest levels between 12:00 h and 15:00 h [36]. Thus, the *W. bancrofti* microfilaria chitinase PCR might not be able to detect MF counts at very low levels. Indeed, the CFA+ MF+ control sample which had only 200 MF/mL tested negative in the *W. bancrofti* microfilarial chitinase qPCR (Table 2).

How would this limitation have affected our study results? We assume that the 12 individuals with positive PCR for MF were just “the tip of the iceberg”—the individuals with highest MF load—but this cannot be proven. However, even with this low number of MF positive cases, we clearly measured a significantly increased HIV susceptibility of the filarial-infected MF positive participants. Adjusting the analysis for known HIV risk factors to prevent confounding did not change this result. However, we could not differentiate whether the previously published increased HIV incidence among Wb-infected individuals was caused by the MF positive subgroup alone, or if both the adult worm and the offspring contributed to this association. It would be interesting to repeat this study with improved filarial diagnostic to confirm or reject our findings. However, we would have to find another study area to do this with a less powerful NTD program.

## 5. Conclusions

In conclusion, this study reveals that *W. bancrofti*-infected individuals with microfilaremia have a dramatically elevated susceptibility towards HIV. In this subgroup of Wb-infected individuals, the HIV susceptibility exceeds the previously described 2.3-fold increased risk for HIV seen in the larger cohort of all Wb-infected individuals (regardless of MF status). Compared to filarial-uninfected persons, the risk of acquiring HIV is approximately 10–12-times higher. If both Wb-infected subgroups are compared, the patent infection is associated with a 4.58-times higher HIV incidence than the latent Wb infection. Further studies should decipher the mechanisms that are responsible for the increased risk of HIV in microfilaria positive individuals and whether anti-filarial treatment in LF endemic areas can reduce the HIV incidence in filarial-infected people.

## Figures and Tables

**Table 1:** Testing of samples with known filarial species and MF count for chitinase PCR sensitivity and specificity measurement.

Nematode Species	N	CFA	*W. bancrofti* MF Count/mL	MF PCR Pos	MF PCR Neg
*W.* *bancrofti*	1	pos	200	0	1
*W.* *bancrofti*	1	pos	1740	1	0
*W.* *bancrofti*	1	pos	2030	1	0
*W.* *bancrofti*	1	pos	2170	1	0
*W.* *bancrofti*	1	pos	2420	1	0
*W.* *bancrofti*	1	pos	4090	1	0
*W.* *bancrofti*	1	pos	4360	1	0
*Mansonella* *perstans*	20	neg	NA	0	20
*Loa* *loa*	6	neg	NA	0	6
*Onchocerca* *volvulus*	20	neg	NA	0	20
not infected *	30	neg	0	1	29
*not infected ^#^*	50	neg	Not determined	0	50

N = count; CFA = circulating filarial antigen; * samples from a Wb endemic area, tested for CFA and MF; ^#^ samples from the Tanzanian study village, tested for CFA.

**Table 2:** Description of Kyela study participants with CFA positive samples.

	MF PCR Performed	No PCR Performed	
Characteristic	MF PCR Tested	MF Not Tested	*p*-Value *
Number of samples	350	156	
Sex (%)			
female	170 (48.6%)	75 (48.1%)	0.918
male	180 (51.4%)	81 (51.9%)	
Age group (years)			
<25	117 (33.2%)	64 (41.0%)	
25–<45	110 (31.5%)	44 (28.2%)	0.257
≥45	123 (35.2%)	48 (35.2%)	
Median age in years (IQR ^$^)	32.7 (21.8–56.7)	28.4.8 (18.7–51.0)	0.090
SES rank, median (IQR)	3.04 (0.86–5.84)	3.93 (1.35–6.14)	0.088

* *p*-value was calculated using Chi-square for binary variables (sex, age group) and Mann–Whitney test for continuous variables (age, SES); ^$^ IQR: interquartile range.

**Table 3:** Uni- and multivariable analysis showing the association between HIV and MF chitinase gene positivity among *W. bancrofti*-infected individuals.

				Univariable			Multivariable		
Covariate	Exp.	N-Pos	Incidence per 100 PY	IRR	95% CI	*p*-Value	IRR	95% CI	*p*-Value
**All** **MF at survey 1**	1109	22	1.98						
Neg *	1070	19	1.78	1.00			1.00		
Pos	39	3	7.75	**4.29**	**(1.33 to 13.9)**	**0.015**	**4.58**	**(1.37 to 15.4)**	**0.014**
**Gender**									
Female *	537	11	2.05	1.00	-	-	1.00	-	-
Male	572	11	1.92	0.94	(0.41 to 2.18)	0.885	0.90	(0.39 to 2.07)	0.796
**Age**									
14–<25 *	312	6	1.92	1.00	-	-	1.00	-	-
25–<45	338	10	2.61	1.35	(0.49 to 3.75)	0.560	1.46	(0.54 to 3.95)	0.453
≥45	413	6	1.45	0.76	(0.25 to 2.36)	0.636	0.75	(0.25 to 2.12)	0.530
**SES rank (per unit)**	-	-	-	0.91	(0.78 to 1.06)	0.213	0.89	(0.76 to 1.04)	0.134

Exp. = exposure time in person years; N-pos = number of positives; PY = person years; IRR = incidence rate ratio; 95% CI = 95% confidence interval; * reference stratum.

**Table 4:** Uni- and multivariable analysis showing the association between HIV and LF status (CFA and MF combined) when adjusted for commonly known HIV risk factors.

				Univariable			Multivariable		
Covariate	Exp.	N-pos	IR per 100 PY	IRR	95% CI	*p*-Value	IRR	95% CI	*p*-Value
**All**	3187	38	1.19						
**Gender**									
Female *	1642	22	1.34	1.00	-	-	1.00	-	-
Male	1546	16	1.04	0.77	(0.41 to 1.48)	0.436	0.55	(0.42 to 1.24)	0.148
**Age**									
14–<25 *	1337	10	0.75	1.00	-	-	1.00		
25–<45	964	18	1.87	2.48	(1.14 to 5.39)	0.022	1.73	(0.66 to 4.52)	0.263
≥45	886	10	1.13	1.51	(0.63 to 3.62)	0.361	1.27	(0.45 to 3.59)	0.657
**LF status**									
CFA neg *	2101	16	0.76	1.00	-	-	1.00	-	-
CFA + MF neg	1051	19	1.81	**2.36**	**(1.21 to 4.59)**	**0.012**	**2.12**	**(1.71 to 4.20)**	**0.030**
CFA + MF pos	35	3	8.69	**11.1**	**(3.43 to 36.2)**	**<0.001**	**12.7**	**(3.48 to 46.2)**	**<0.001**
**Marital status**									
Never married *	1113	7	0.63	1.00	-	-	1.00	-	-
Married	1357	19	1.40	2.22	(0.93 to 5.29)	0.073	0.94	(0.27 to 3.22)	0.922
Separated ^#^	718	12	1.67	2.64	(1.04 to 6.73)	0.042	1.20	(0.35 to 4.10)	0.772
**No. of sex partners**									
None *	285	2	0.70	1.00	-	-	1.00	-	-
One	1487	20	1.35	1.91	(0.44 to 8.21)	0.386	2.63	(0.51 to 13.7)	0.251
Two or more	378	11	2.91	4.09	(0.90 to 18.6)	0.069	8.52	(1.49 to 48.7)	0.016
No info	1038	5	0.48	0.69	(0.13 to 3.56)	0.656	1.29	(0.24 to 7.01)	0.771
**Circumcised (male)**									
No *	1351	15	1.11	1.00	-	-	1.00	-	-
Yes	195	1	0.51	0.42	(0.06 to 3.19)	0.418	0.48	(0.06 to 3.60)	0.476
N/A (female)	1642	22	1.34	1.00	(omitted)	-	1.00	(omitted)	-
**SES rank (per unit)**	-	-	-	0.92	(0.81 to 1.03)	0.158	0.91	(0.80 to 1.03)	0.146

Exp. = exposure time in person years; N-pos = number of positives; IR = incidence rate, PY = person years; IRR = incidence rate ratio; 95% CI = 95% confidence interval; * reference stratum; ^#^ separated, divorced or widowed.

## Data Availability

The data presented in this study are available on request from the corresponding author. The data used for this analysis contain the GPS positions of all participating households, which could be used to find and identify participating households and individuals. Thus, making them publicly available would constitute a severe breach of confidentiality.
